# Coflowering invasive plants and a congener have neutral effects on fitness components of a rare endemic plant

**DOI:** 10.1002/ece3.7375

**Published:** 2021-03-20

**Authors:** Diane L. Larson, Jennifer L. Larson, Amy J. Symstad, Deborah A. Buhl, Zachary M. Portman

**Affiliations:** ^1^ Northern Prairie Wildlife Research Center U.S. Geological Survey Hot Springs AR USA; ^2^ Department of Entomology University of Minnesota St. Paul MN US

**Keywords:** endemic plant, *Eriogonum* Michx, halictid bees, invasive plants, *Melilotusofficinalis* (L.) Lam, pollination ecology, *Salsolatragus* L

## Abstract

Network analyses rarely include fitness components, such as germination, to tie invasive plants to population‐level effects on the natives. We address this limitation in a previously studied network of flower visitors around a suite of native and invasive plants that includes an endemic plant at Badlands National Park, South Dakota, USA. *Eriogonum visheri* coflowers with two abundant invasive plants, *Salsola tragus* and *Melilotus officinalis*, as well as a common congener, *E. pauciflorum*. Network analyses had suggested strong linkages between *E. visheri* and *S. tragus* and *E. pauciflorum*, with a weaker link to *M. officinalis*. We measured visitation, pollen deposited on stigmas, achene weight and germination over three field seasons (two for germination) in four populations (two in the final season) of *E. visheri* and applied in situ pollen treatments to *E. visheri*, adding pollen from other flowers on the same plant; flowers on other *E. visheri* plants; *S. tragus, M. officinalis*, or *E. pauciflorum*; open pollination; or excluding pollinators. Insect visitation to *E. visheri* was not affected by floral abundance of any of the focal species. Most visitors were halictid bees; one of these (*Lasioglossum packeri*) was the only identified species to visit *E. visheri* all three years. Ninety‐seven percent of pollen on collected *E. visheri* stigmas was conspecific, but 22% of flowers had >1 grain of *E. pauciflorum* pollen on stigmas and 7% had >1 grain of *S. tragus* pollen; <1% of flowers had *M. officinalis* pollen on stigmas. None of the pollen treatments produced significant differences in weight or germination of *E. visheri* achenes. We conclude that, in contrast to the results of the network analysis, neither of the invasive species poses a threat, via heterospecific pollen deposition, to pollination of the endemic *E. visheri*, and that its congener provides alternative pollen resources to its pollinators.

## Graphical abstract

*Eriogonum visheri* (Visher's buckwheat) is a rare endemic plant of management concern in the northern Great Plains. Two invasive species, *Melilotus officinalis* (yellow sweetclover) and *Salsola tragus* (Russian thistle) and an abundant congener (*E. pauciflorum*; fewflower buckwheat) occur and coflower with *E*. *visheri*, suggesting that they might interfere with pollination of the rare species. We found no evidence of pollination interference by either of the invasive species. In addition, evidence suggests that the abundant congener may provide an alternative resource to support pollinators that also visit the rarer species.

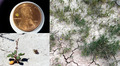

## 1 INTRODUCTION

Competition for pollinator services, recognized by Darwin, has been evaluated with respect to visitation frequency, quality of pollen received, and fitness outcomes (Spellman et al., 2015; Thijs et al., 2012) for the competing plant species. With the realization that invasive plants can have a greater impact than habitat fragmentation on flower visitation networks (Hansen et al., 2018), understanding effects of such disruption for fitness of native plants has become imperative. Detrimental effects of non‐native plant invasion on native plant fitness is not a foregone conclusion, however. Studies show a range of effects from facilitative to neutral to competitive, often depending on the density, taxonomy, or floral morphology of the invader (Bruckman & Campbell, 2016; Iler & Goodell, 2014; Molano‐Flores, 2014; Sun et al., 2013).

Locally rare or endemic plant species may be especially vulnerable to effects of non‐native invasive species if the invaders reduce opportunities for outcrossing or result in high levels of interspecific pollen transfer. On the other hand, invading plants with abundant flowers may draw additional pollinating insects to the area that augment pollen movement among the endemic flowers (Jakobsson et al., 2009). Effects on endemic plant fitness would depend on the fidelity of individual insects to their flowers and impacts of nonconspecific pollen that may be deposited on the endemic plants’ stigmas. Nonconspecific pollen may clog the stigmatic surface (Carvallo & Medel, 2016; Tscheulin & Petanidou, 2013) or may be allelopathic to conspecific pollen (Ashman & Arceo‐Gomez, 2013).

Endemism is uncommon among plants in the Great Plains, with the exception of those specific to habitat “islands” with unusual edaphic characteristics, such as those preserved in Badlands National Park (BNP), South Dakota, USA. The clay outwash from the largely unvegetated erosional features, known as “badlands sparse vegetation complex” (Von Loh et al., 1999), harbors a handful of rare and/or endemic plant species, including *Eriogonum visheri* A. Nelson (Visher's buckwheat), the focus of the current study. *Eriogonum visheri* is a species of concern to management agencies in the northern Great Plains and has been assigned the rank of G3, which indicates globally vulnerable species, by NatureServe (Ladyman, 2006). Primary threats to the species are thought to be habitat destruction and invasion by exotic plants; little is known about the specifics of its habitat requirements, as populations often occur in isolated patches unevenly distributed across expanses of seemingly appropriate habitat (Ladyman, 2006).

Two invasive plant species with the potential to interfere with pollination of the buckwheat, *Salsola tragus* L. (Russian thistle) and *Melilotus officinalis* (L.) Lam. (yellow sweetclover), were identified in a study of pollination networks around *E. visheri* at BNP (Larson et al., 2014). *Salsola tragus* was determined to be more likely to have an effect on pollination of the endemic buckwheat based on its occurrence within the same module (i.e., groups of plants and flower visitors that interact more with each other than with those outside the module (Olesen et al., 2007)) as *E. visheri*. *Melilotus officinalis* shared very few flower visitors with *E. visheri* and occupied a different module in each of the two years of the study, so was deemed a lesser threat (Larson et al., 2014). Left unresolved by that study were fitness‐level effects of these two species on *E. visheri*, for which, as an annual, production of viable seed is crucial. We undertook the present study to examine if the presence of either of the invasive species or a common, co‐occurring congener, *Eriogonum pauciflorum* Pursh (fewflower buckwheat), that shared a module with *E. visheri* in one of the years of the earlier study resulted in changes to seed viability via direct effects of pollen on *E. visheri* stigmas, or indirect effects via visitation.

To determine this, we asked the following questions: (a) Are insect species that visit *E. visheri* consistent among years and equally likely to carry pollen from *E. visheri* and other species? Is visitation related to flower abundance at a site? (b) How common is the presence of nonconspecific pollen on *E. visheri* stigmas? (c) Does presence of *E. pauciflorum, S. tragus*, or *M. officinalis* pollen on stigmas of *E. visheri* influence achene weight or germination likelihood? Our overall goal was to determine whether results of this study are consistent with those of the network analysis, especially with respect to management considerations.

## 2 METHODS

### 2.1 Study species and study sites

**FIGURE 1:**
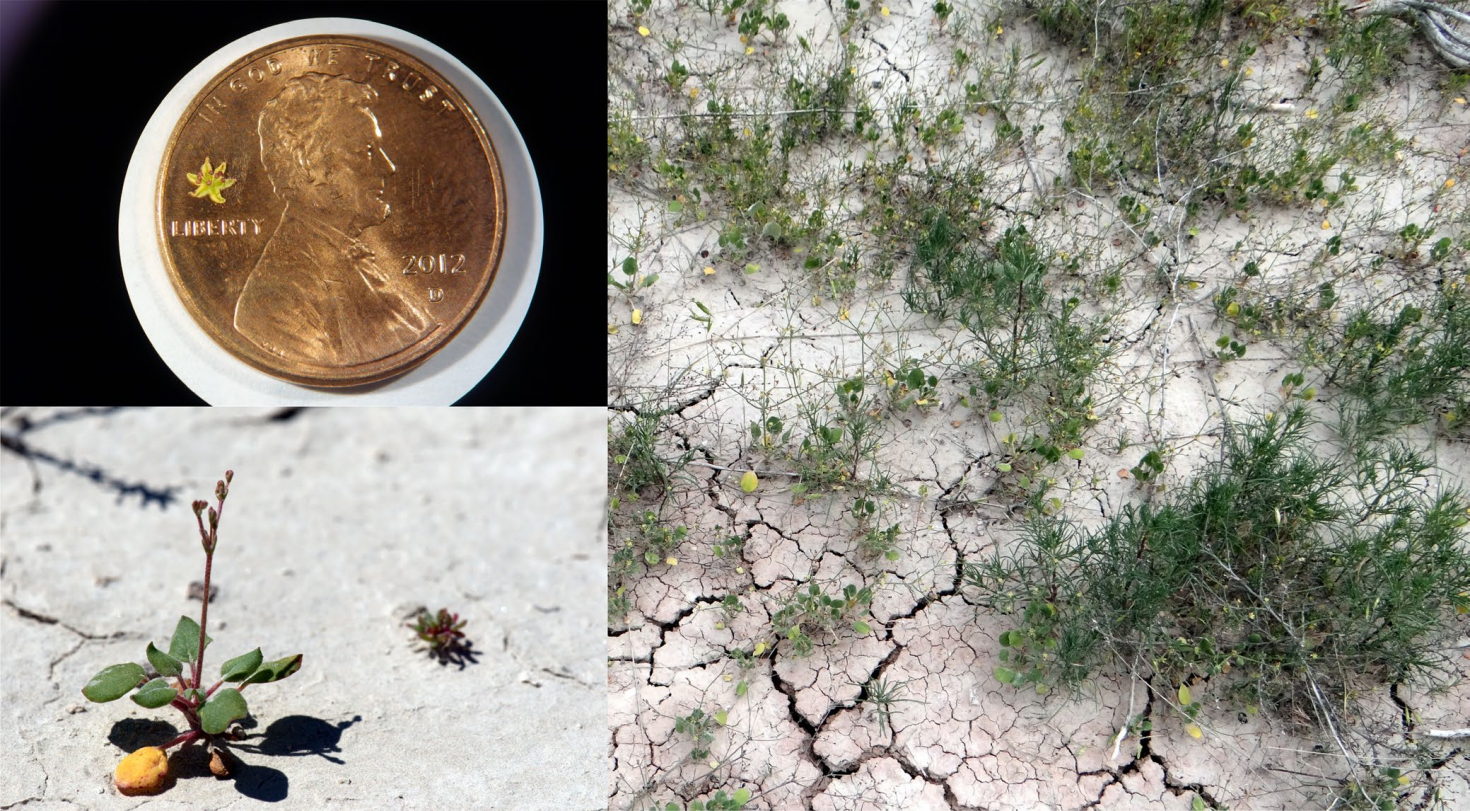
Counterclockwise from upper left, *Eriogonum visheri* flower with a U.S. penny for scale (photograph by Mary Behlke); *E. visheri* rosette just beginning to bolt (photograph by Diane Larson); *E. visheri* with *Salsola tragus* (lower right) on cracked clay soils typical of *E. visheri* habitat at Badlands National Park (photograph by Diane Larson)

*Eriogonum visheri* (Figure 1) is a summer annual species in the Polygonaceae restricted to sparsely vegetated clay outwash soils found in badlands habitats in South Dakota, North Dakota, and Montana (Ladyman, 2006). Rosettes form in June, and flowering begins in July and may continue into September at BNP if moisture is adequate. The tiny (~2 mm diameter) yellow flowers are protandrous and may close in the evening but open again the following day. The total duration of a single flower is unclear but can exceed two days at our study sites. Closure brings the dehiscent anthers in close contact with the stigmas, suggesting that the species is likely autogamous. Flowers may occur singly or in groups of up to 4 per involucre. Flower visitors to *E. visheri* include small halictid bees, beeflies, ants, and wasps (see Tables S1 and S2 in Larson et al., 2014), from which we infer that floral rewards include both nectar and pollen.

*Eriogonum pauciflorum*, a mat‐forming perennial, is common throughout the badlands sparse vegetation complex at BNP. The white‐to‐pinkish flowers occur in a single cluster at the top of relatively long stems. Flowering begins in June and may continue through August (Great Plains Flora Association, 1986). *Eriogonum pauciflorum* commonly occurs interspersed with *E. visheri* at BNP and attracts many of the same flower visitors (Larson et al., 2014).

*Salsola tragus* is an annual species in the Chenopodiaceae introduced to South Dakota from Eurasia as a contaminant of crop seed in 1873 (Beckie & Francis, 2009; Great Plains Flora Association, 1986). It typically is much taller (up to a meter in height) and produces more flowers than either of the native *Eriogonums* with which it grows intermingled. Flowering typically occurs in August—October (Great Plains Flora Association, 1986). Though often thought to be wind‐pollinated, the bright yellow anthers were found to be visited by a variety of colletid and halictid bees in the southwestern US (Blackwell & Powell, 1981) as well as in BNP (Larson et al., 2014).

*Melilotus officinalis* is a biennial species in the Fabaceae and may grow even taller than *S. tragus* (0.5–2 m). It was first recorded at BNP in 1959 (Lindstrom, 1959) and occurs throughout the park, though with large inter‐annual variation in abundance. The yellow flowers are attractive to honeybees (*Apis mellifera*; Otto et al., 2017); flowering typically occurs during June and July at BNP.

**FIGURE 2:**
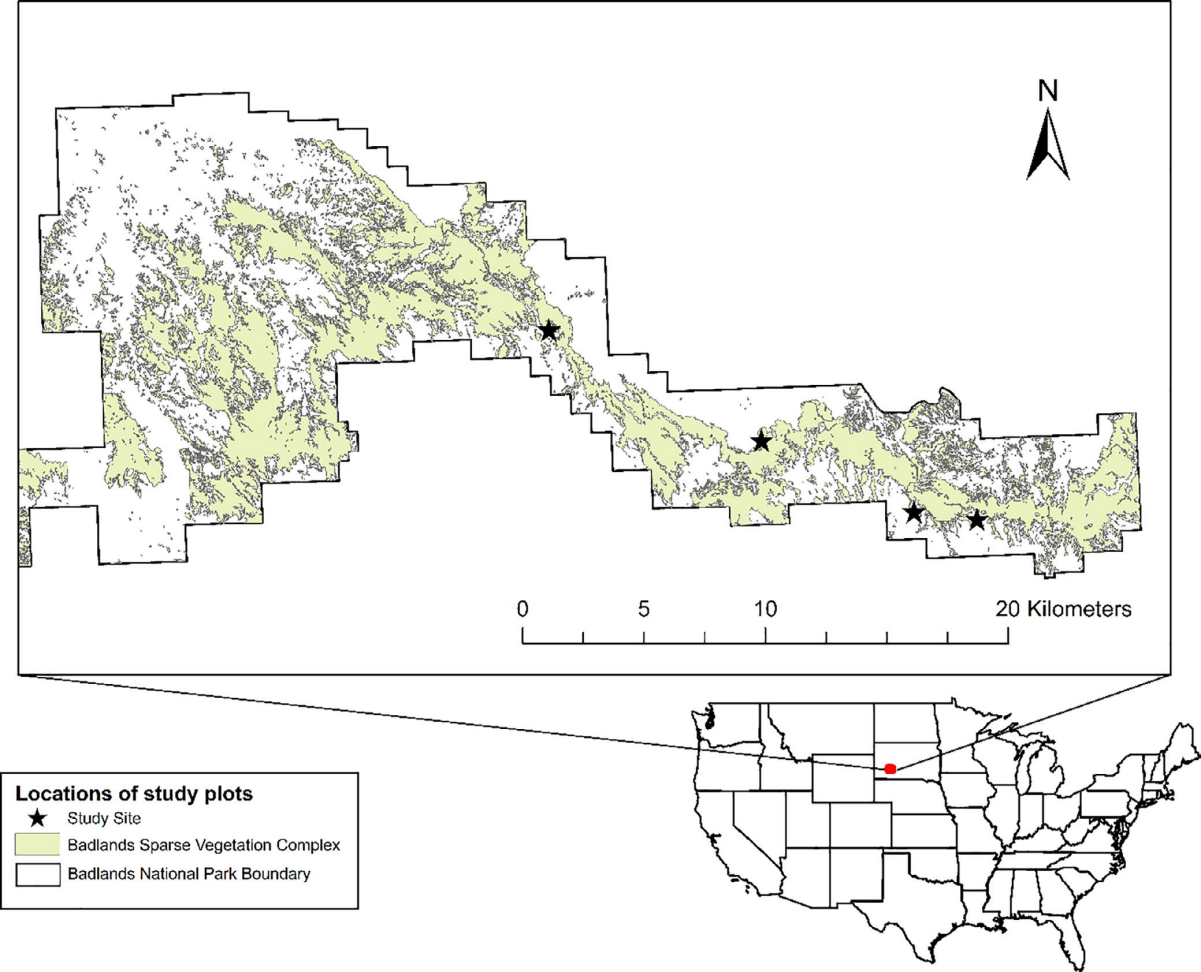
Map of *Eriogonum visheri* study sites at Badlands National Park, *SD*, USA. The two closest study sites were separated by 2.6 km. Note that the extent frame shows only the NE section of the park

This study occurred in 2014, 2015, and 2017. We used two of the four sites from the previous study (Larson et al., 2014); one no longer had *E. visheri* and one was inaccessible at the beginning of the current study. To find two replacement sites, we consulted BNP records of prior locations of *E. visheri* and prioritized those that were within an hour's walk of the access road through the park. All four sites we used in this study were sufficiently distant from each other to preclude sharing of insect pollinators (Figure 2). All study sites were located in badlands sparse vegetation and contained >100 individuals of *E. visheri* in 2014. Each study site was 1‐ha centered on the *E. visheri* population. In 2017, reduced populations of *E. visheri* and limited funding caused us to focus on the two sites with adequate numbers of plants to accommodate pollen treatments.

Growing‐season (April—September) precipitation at BNP was 377, 515, and 247 mm in 2014, 2015, and 2017, respectively. Growing‐season mean maximum temperature was 25.9°C, 26.7°C, and 27.8°C in 2014, 2015, and 2017, respectively (NOAA Climate Data Online, https://www.ncdc.noaa.gov/cdo‐web/datasets#GHCND. Downloaded 2021–01–07).

### 2.2 Flower abundance

We spaced ten 75‐m long, variable‐width transects equidistantly within each 1‐ha site. We counted the number of flowering forbs by species (all species in flower were included) on each transect approximately every two weeks. Transect width varied between 0.25 and 2.0 m; very abundant flowers were counted within narrower transects than were uncommon flowers. All counts were standardized to 2‐m width for analysis. Methods followed Larson et al., (2014).

### 2.3 Insect visitation

We delineated insect visitation plots with pieces of PVC tubing. Depending on the configuration of *E. visheri* flowering plants, the plot could be 0.5 m × 2 m or 1 m × 1 m. Because our aim was to include as many flowering plants as possible, plot location varied from survey to survey within a day and from day to day at any study site. No more than three visitation plots per site were conducted in a single day. We observed plots for 20 min, plus added handling time for captured insects. Any insect observed to visit an *E. visheri* flower (i.e., it was in contact with the reproductive parts of a flower) was recorded on a data sheet; insects were only captured if it was possible to do so without harming the *E. visheri* plant. We placed captured insects individually in vials charged with ethyl acetate. When quiet, we transferred the insect to a glassine envelope labeled with a unique identifier and information on the location and time of capture, then placed the envelope in a jar also charged with ethyl acetate. Although we recorded type of insect on the data sheet, comparison with those identified after capture suggests that small bees and flies were often confused. We therefore only analyzed the observational data collectively as insect visits, rather than within taxa.

To learn which species of pollen insects carried, we removed pollen from the bodies, including the surface of the scopae (Parker et al., 2015), of captured insects using small cubes of fuchsin jelly (Kearns & Inouye, 1993) as described in Larson et al., (2014). Cubes were gently melted on slides, covered with a cover slip, and the border of the slip then painted with latex paint to protect the slide contents. Pollen was identified to species and counted at 10 – 100x with a light microscope; fewer than 10 grains of a plant species from an individual insect was considered contamination because small amounts of pollen could have been picked up from the net.

Bees were identified by Z. Portman using published keys and revisions: *Agapostemon* (Roberts, 1972), *Halictus* (Roberts 1973), *Lasioglossum* (Gibbs, 2010, 2011), and *Melissodes* (LaBerge, 1961). Specimens are deposited in the BNP museum collection. Other insects were identified only to order, with the exception of the fly, *Paragus haemorrhous*, which was distinctive and quite abundant.

### 2.4 Achenes from pollen treatments

At each site, we marked 90–100 *E. visheri* rosettes in June, prior to bolt, each year, then randomly assigned them to pollination treatments, one treatment per plant (Table 1). Because plants were assigned treatments before flowers had formed, one treatment (MOP in 2015) did not have enough surviving treatment plants for some analyses. We collected pollen on wooden toothpicks per the assigned treatment and applied it to the *E. visheri* stigma. To be sure we were collecting enough pollen, we examined a sample of toothpicks for the targeted pollen under a microscope. In 2014, to identify which flowers were treated on each plant, we used varying numbers of knots tied into thread fixed to the branch proximal to the flower(s) to be treated. We secured small mesh bags around the treated branch. Because flowers were borne in groups of up to 4 and could not be individually marked, we could not be certain which flower(s) had been previously treated; therefore, all open flowers in the group were treated at each visit. Although we kept track of the number of flowers treated, we could not be sure that every flower in every marked group was treated, as they opened and closed repeatedly and may never have been open during site visits.

**TABLE 1:** Pollen treatments and the years in which they were applied to *Eriogonum visheri* plants at Badlands National Park study sites

Treatment	2014	2015	2017
EVP: *E. visheri* pollen from 3 flowers from 3 different plants added	X	X	X
WEP: *E. visheri* pollen from 3 flowers on the same plant added	X		
MOP: *E. visheri* + *M. officinalis* pollen from 3 flowers from 3 different plants added	X	X	
STP: *E. visheri* + *S. tragus* pollen from 3 flowers from 3 different plants added		X	
EPP: *E. visheri* + *E. pauciflorum* pollen from 3 flowers from 3 different plants added		X	
OP: open pollinated	X	X	X
PE: pollinators excluded	X		X

In 2015 and 2017, we modified our methods because the mesh bags on individual branches often caused the branches to break. When flowers began to open, we covered entire plants in mesh bags, except those plants in the open‐pollinated treatment. We applied pollen treatments to every open flower each time a plot was visited (approximately every 2–3 days). This resulted in a small, but unknown, number of flowers inevitably being missed.

As achenes formed, we checked for ripeness by touching the achene with the tip of a pencil; ripe achenes easily dehisced. We did not forcefully remove achenes. Most achenes that dehisced were caught in the mesh bag, which we carefully opened at each visit after placing a white cloth around the base of the plant to catch the achenes that fell out of the bag. We bagged open‐pollinated plants when we saw no new flowers on the plant, which corresponded with the beginning of achene ripening. When all achenes on a plant had dehisced and the plant appeared senescent, the plant was collected, dried, and weighed to document any systematic differences in plant growth among treatments.

Although we did track and collect aborted flowers and achenes (Table S1), the minute flowers and fragile plants made it impossible to mark and follow the fate of individual flowers and achenes under field conditions. Thus, our methods almost certainly underestimated counts of aborted flowers and aborted achenes, making it impossible to estimate seed set per plant. Consequently, we focused our analyses on differences in achene weight and germination of filled (i.e., not aborted) achenes among treatments.

**FIGURE 3:**
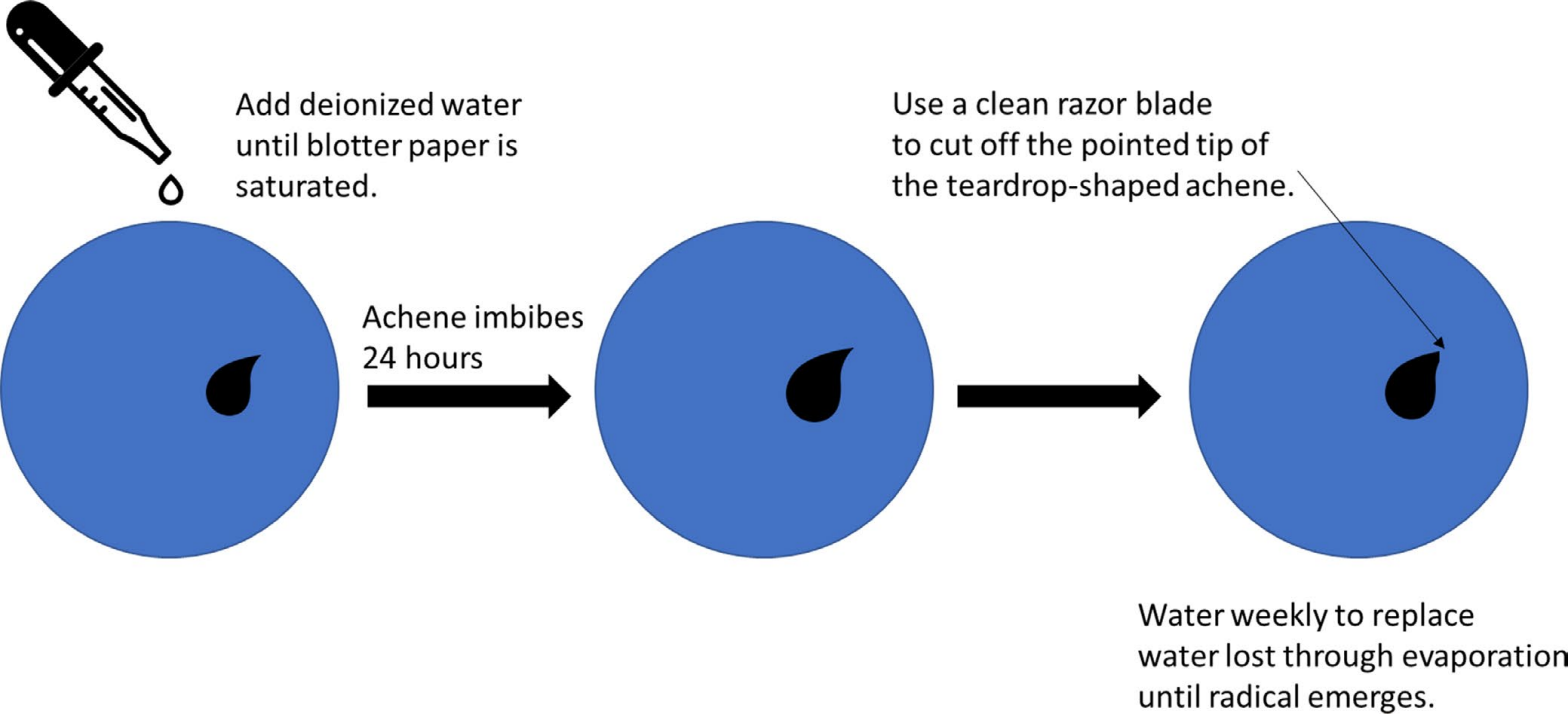
Germination protocol for *Eriogonum visheri* achenes. Blotter paper fits into the bottom of covered petri dishes

Following field collection, achenes were kept refrigerated at 2.5°C as a cold pretreatment for up to one year before germination procedures began. Achenes collected in 2014 were used to establish a successful germination protocol. We weighed each achene individually and placed it on 2‐inch blue blotter paper (Anchor Paper Company St. Paul, MN) in a 2‐inch clear plastic petri dish (Fisherbrand™); we then wetted the paper with deionized water until slightly past saturation and sealed the plates with Parafilm® for 24 hr to allow the achenes to imbibe. Following the 24‐hr period, we cut the very tip off of the achenes (Figure 3), returned them to the petri dish and resealed it. This scarification step was deemed necessary based on 2014 trials in which imbibing alone (as suggested in Meyer & Paulsen, 2000 for other species of *Eriogonum*) resulted in a <4% germination rate. Petri dishes were placed in a greenhouse under 12 hr light at ~ 75˚F and 12 hr dark at ~ 55˚F. We counted and removed achenes with a >1 mm radical (indicating germination) weekly and excluded those that developed mold prior to radical growth from the analysis.

### 2.5 Pollen on stigmas

We collected no more than four (typically 1–2) flowers from an *E. visheri* plant on any given day. Collection was haphazard, but an attempt was made to sample all plants at each site. In 2014, most of the collections happened within a single week, after which flower availability declined; collections in 2015 were more evenly distributed, but the season was again short in 2017 (Table S2). Flowers were removed from the plant with forceps and placed individually into glassine envelopes labeled with site, date, collector, and plant ID.

Individual flowers were kept in their own glassine envelopes prior to processing. Each flower was dissected on a cube of fuchsin jelly on a glass slide using a Meiji EMZ dissecting scope; each of 3 stigmas was separated onto the jelly cube. We gently heated the fuchsin jelly until it melted, then placed a cover slip on top. We scanned the entire area of melted jelly for pollen grains using a Leica DMLS microscope at 10 – 100x, and recorded pollen to the species‐level when possible; pollen grains were compared to a pollen reference collection made at the study sites. It is important to note that it was not possible to distinguish *E. visheri* pollen that originated from the flower's own anthers from that brought to the stigma by an insect pollinator. The minute flowers were impossible to emasculate in the field. Data were summarized as number of grains by species per stigma.

### 2.6 Statistical analyses

#### 2.6.1 Flower counts

We used negative binomial regression models in PROC GLIMMIX (SAS Institute Inc. 2018; SAS Institute Inc. (c) 2002–2012) to test for differences in flower counts per site among years. Differences were tested for *E. visheri, E. pauciflorum, S. tragus*, and *M. officinalis*. In the model for each species, site was included as a random block and repeated samples each year were treated as subsamples in time. We also tested for differences in total flower counts (separately including and excluding *M. officinalis* because the huge number of *M. officinalis* flowers in 2014 obscures any differences in the other species) using similar models but assuming a lognormal distribution.

#### 2.6.2 Pollen on insects

To assess if the amount of pollen carried by the captured insects is related to flower abundance, we first screened the data to determine which insect species were captured in sufficient numbers and carried sufficient *E. visheri* pollen for statistical analysis. For those species, we ran negative binomial regressions in PROC GLIMMIX (SAS Institute Inc. 2018; SAS Institute Inc. (c) 2002–2012) to assess the relationship between the amount of *E. visheri* pollen an insect carried and the number of *E. visheri* flowers present. Analyses were carried out within years.

#### 2.6.3 Effects of pollen treatments

We used generalized linear mixed models (GLMM) to examine differences in response variables among pollen treatments. Three response variables were considered: plant weight, achene weight, and germination. In each of these, individual plant was the sample unit. We assumed a lognormal distribution for plant weights since observed plant weight values were skewed. A normal distribution was assumed for achene weight and germination was a binomial variable. Models were run separately for each year. The model was a randomized block design with complete blocks in 2014 and 2017 but incomplete blocks in 2015. For plant weight, the model included site and site*treatment as random effects. For achene weight and germination, the model included site, site*treatment, and plant within site and treatment as random effects. The relationship between the response variables and collection date was assessed using analysis of covariance methods (Milliken & Johnson, 2002) to determine if the covariates were related to each response and if the relationship was the same for all treatments (common slopes) or different for each treatment (different slopes). For germination, achene weight also was included as a possible covariate along with collection date. After the correct form of each covariate was determined, differences in treatment least squares means were tested while accounting for the covariates.

When compared with open‐pollinated (OP) or supplemental *E. visheri* pollen (EVP) treatments, germination rate for the pollinator exclusion treatment (in 2017; Table 1) tests for the extent of pollen limitation due to autogamy in *E. visheri*. We used a modification of equation 2 in Baskin and Baskin (2018) to calculate this extent. Baskin and Baskin specify that the open‐pollinated flowers should have supplemental pollen applied. In our study, open‐pollinated flowers had no supplemental pollen, and flowers that received supplemental pollen were bagged because we were interested in effects of pollen from the invasive species and wished to exclude it from these flowers. Our tests for pollen limitation due to autogamy therefore compare germination rates for the pollinator exclusion treatment with (1) open‐pollinated and (2) pollen‐supplemented treatments:

$$\text{PL}=P_{s}‐P_{e}/P_{\text{max}}\left[P_{s},P_{e}\right]$$

where PL refers to pollen limitation due to autogamy, P_s_ is germination rate per achene for either open‐pollinated or supplemental *E. visheri* pollen treatment, and P_e_ refers to germination rate per achene when pollinators were excluded; P_max_ is the larger of P_s_ or P_e_. As noted above, we lack reliable estimates of aborted flowers and achenes, so here we evaluate effects of autogamy only on achenes that dehisced per the protocol described above.

## 3 RESULTS

**FIGURE 4:**
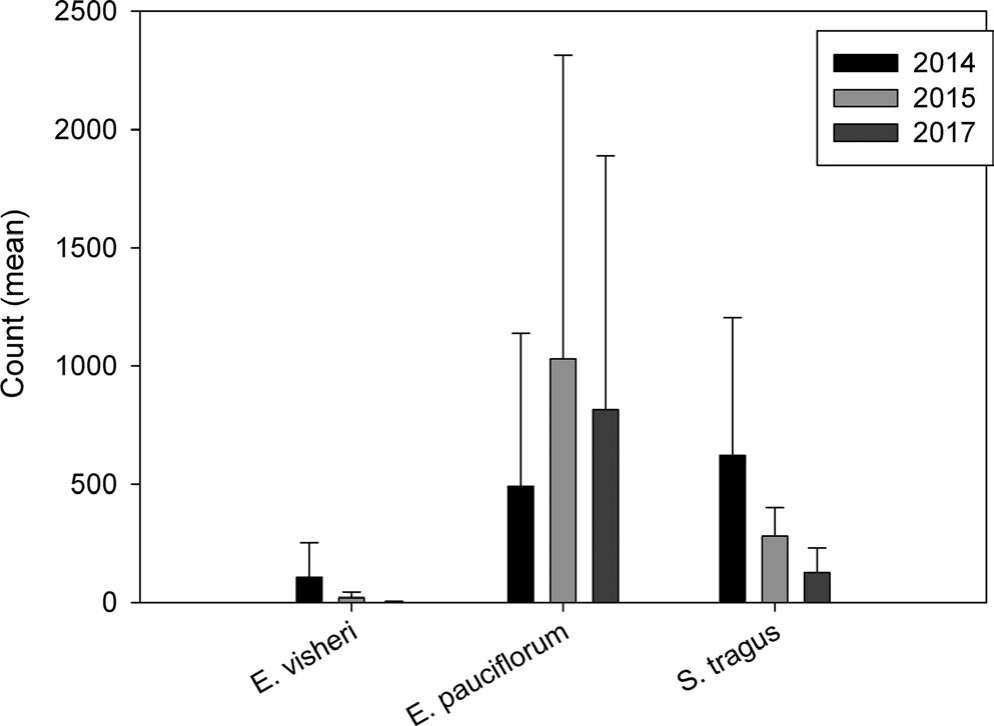
Yearly flower counts/1‐ha site of study species, *Eriogonum visheri, E. pauciflorum, and Salsola tragus*, at the study sites (shown are least square means + SE); *Melilotus officinalis* is not shown here because the numbers in 2014 were 5x greater than the other flower species. See text for *M. officinalis* flower count estimates

*Eriogonum visheri* flowers were marginally more abundant in 2014, the first year of the study (F_2, 4_ = 4.89, *p* =.0843; Figure 4), and *M. officinalis* flowers significantly so in 2014 (F_2, 4_ = 47.84, *p* =.0016; 309,420 + 210,081, 204 + 64, and 633 + 384 (mean total count + SE per 1‐ha site) in 2014, 2015, and 2017, respectively). *Eriogonum pauciflorum* and *S. tragus* flower counts did not vary significantly among years (F_2,4_ = 0.94, *p* =.4617 and F_2, 4_ = 0.82, *p* =.5038, respectively; Figure 4), nor did the mean total flower count, not including *M. officinalis* (F_2, 4_ = 2.36, *p* =.2104; Figure S1a). Mean total flower count including *M. officinalis* was much greater in 2014 than in the other two years (F_2, 4_ = 18.69, *p* =.0093; Figure S1b).

### 3.1 Characterize pollinator visitation to *E. visheri*

In 2014, we observed 67 plots between 2 and 30 July and counted 106 individual insects visiting *E. visheri* flowers (mean 1.58 per plot). Flowering continued for a longer time in 2015, lasting from 17 June to 21 September; we observed 358 insects visiting *E. visheri* on 123 plots (mean 2.91 insects per plot). A short flowering season in 2017, lasting from 4 to 20 July at the two sites we monitored, resulted in 88 insects observed visiting *E. visheri* on 47 plots (mean 1.87 per plot).

**FIGURE 5:**
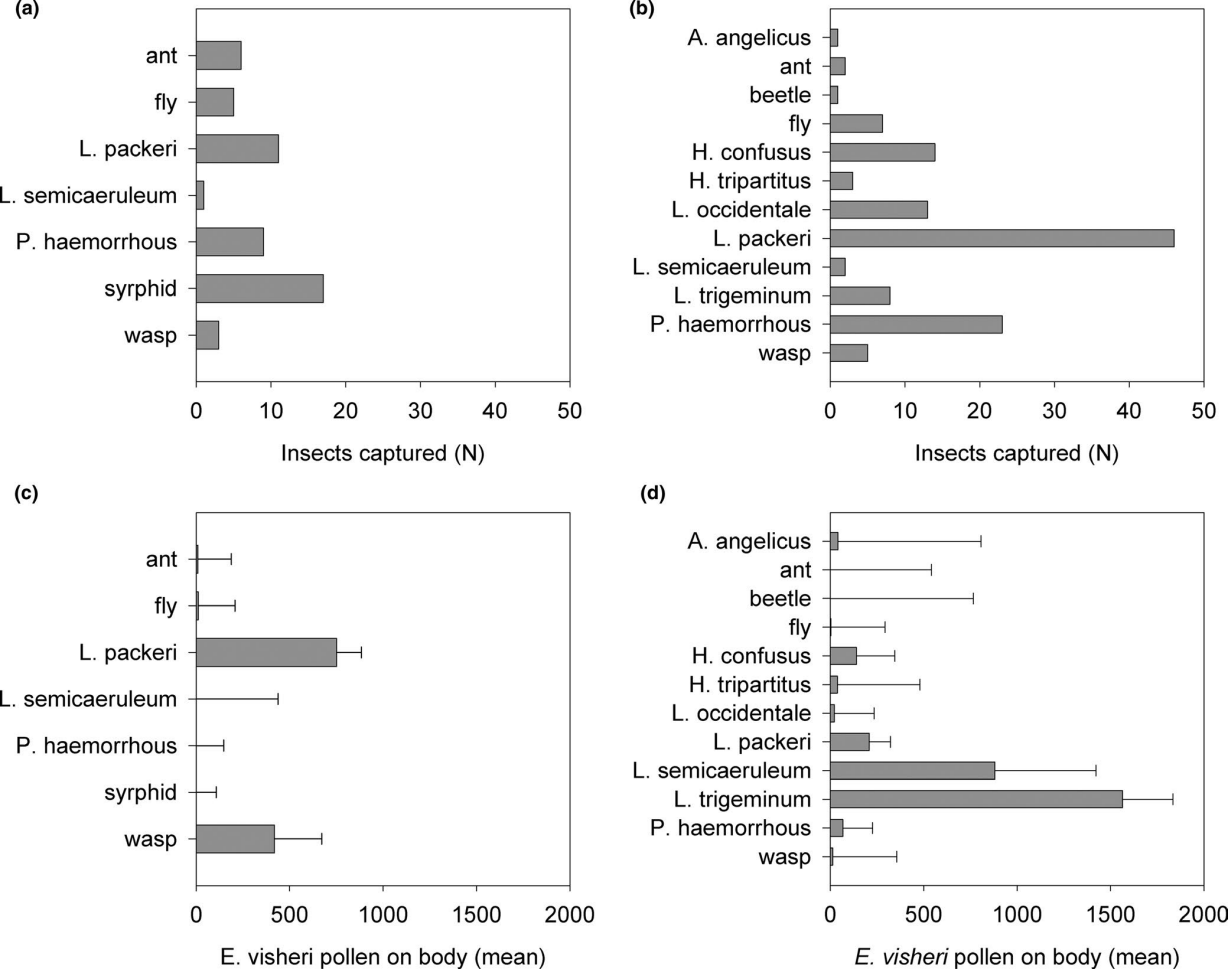
Total captured insects at visitation plots in (a) 2014 and (b) 2015; and mean + SE *Eriogonum visheri* pollen grains removed from insect species captured during visitation plot observations in (c) 2014 and (d) 2015. Insect genera: *Lasioglossum, Paragus, Agapostemon, and Halictus*

Based on captured insects, for which we have positive identification, halictid bees were the most common visitors to *E. visheri* (Figure 5); *Lasioglossum packeri*, a small sweat bee, was the only identified species to visit *E. visheri* each year of the study. Some species, such as *Halictus confusus, H. tripartitus, Lasioglossum occidentale*, and *L. trigeminum*, were captured only in 2015, when flowering lasted more than a month longer than in the other two years.

**FIGURE 6:**
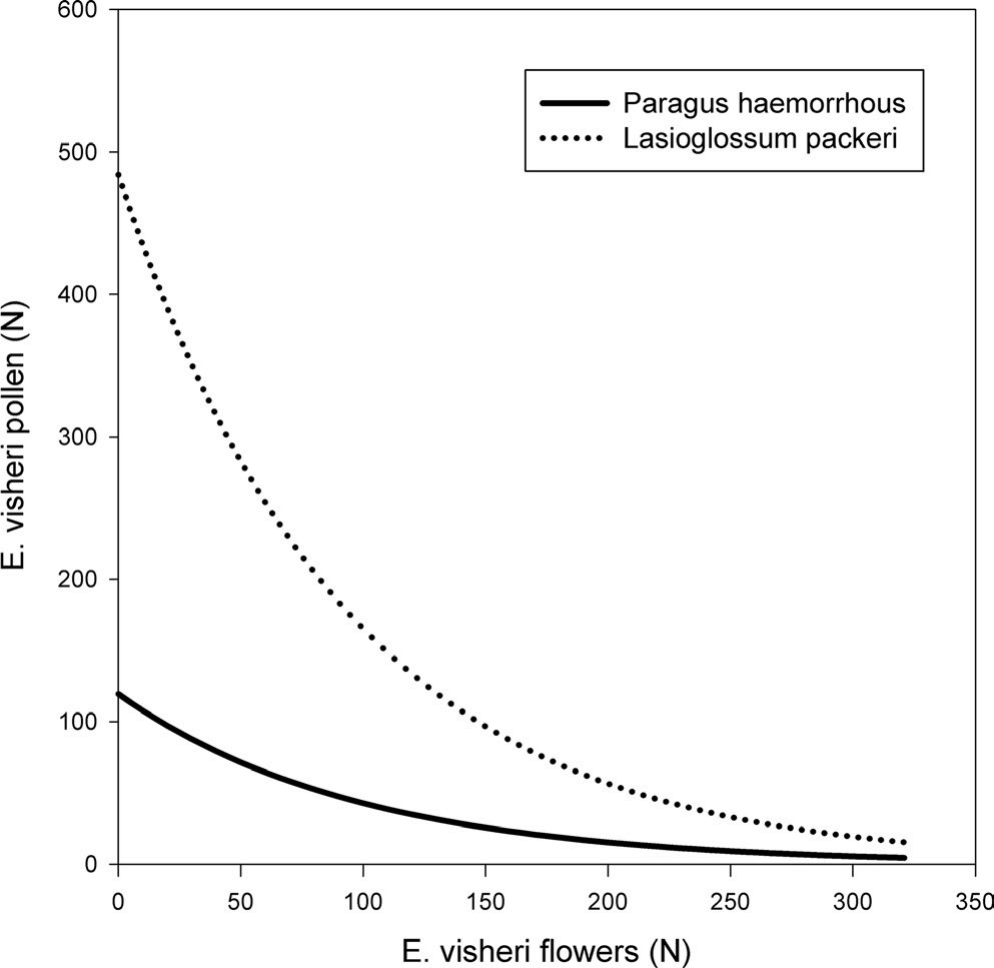
*Eriogonum visheri* pollen carried by the insects *Paragus haemorrhous* and *Lasioglossum packeri* as a function of *E. visheri* flower counts in 2015

The amount of *E. visheri* pollen carried by individual insects captured on *E. visheri* varied greatly among species in 2014 and 2015 (Figure 5). Considering all captured insects across all years, 24% carried only *E. visheri* pollen (43% of these were *L. packeri*), 21.8% carried *E. visheri* plus *S. tragus* pollen; 7.8% carried *E. visheri* plus *E. pauciflorum*. Only one insect (a fly) carried both *E. visheri* and *M. officinalis* pollen and one sweat bee carried only *M. officinalis* pollen (Table S3). Forty‐six percent of captured insects carried no *E. visheri* pollen. Sample sizes per insect species were large enough to examine the relationship between flower availability at sites and *E. visheri* pollen carried on the insects’ bodies for only three species, *H. confusus* in 2015, *L. packeri* in all three years, and *Paragus haemorrhous*, a small hoverfly, in 2015. *Eriogonum visheri* pollen abundance on *H. confusus* in 2015 was unrelated to *E. visheri* flower counts (F_1,12_ = 0.05, *p* =.83); in contrast, *E. visheri* pollen and flower counts were significantly associated for both *P. haemorrhous* and *L. packeri* (F_1, 25_ = 5.51, *p* =.0272 and F_1, 44_ = 6.82, *p* =.0123, respectively). The mean amount of pollen carried by these two insect species declined with increasing number of *E. visheri* flowers counted (Figure 6). The amount of *E. visheri* pollen carried by *L. packeri* in 2014 and 2017 was unrelated to counts of *E. visheri* flowers (F_1, 9_ = 2.95, *p* =.1197 and F_1, 10_ = 1.7, *p* =.2215 for 2014 and 2017, respectively). Note that counts of *E. visheri* flowers were correlated with counts of all flowers in each year (r = 0.42, *N* = 54, *p* =.0017; r = 0.78, *N* = 132, *p* <.0001; and r = 0.74, *N* = 12, *p* =.0062 for 2014, 2015 and 2017, respectively).

### 3.2 How common is the presence of nonconspecific pollen on *E. visheri* stigmas?

Across all years, 97% of pollen counted on all slides of crushed *E. visheri* stigmas was conspecific; 32% of stigma slides (across all years) contained at least 1 other pollen species. Of the 807 slides examined (one slide corresponds to one flower's collected stigmas), 58 contained at least 1 grain of *S. tragus* pollen, 7 contained at least 1 grain of *M. officinalis* pollen, and 185 contained at least 1 grain of *E. pauciflorum* pollen. Twenty‐seven had no pollen of any species. (See data from which these summaries were calculated in Larson et al. (2021)).

### 3.3 Does presence of *E. pauciflorum*, *S. tragus*, or *M. officinalis* pollen on stigmas influence achene weight or germination likelihood?

**FIGURE 7:**
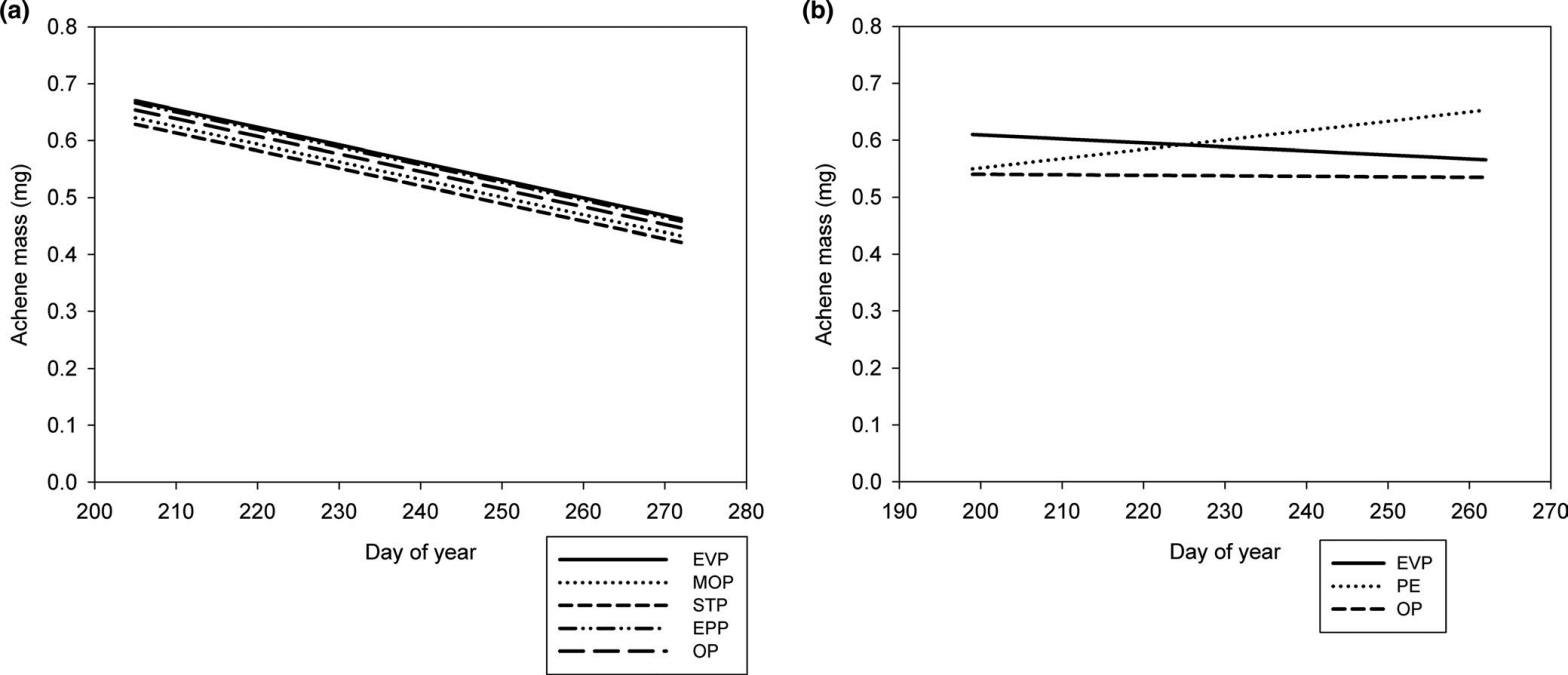
Achene weight of *Eriogonum visheri* as a function of collection date (day‐of‐year) and treatment in (a) 2015 and (b) 2017. Abbreviations for treatments in the legends follow Table 1. Achene weight did not vary among pollen treatments in either year. Achene weight tended to increase over time in the pollinator exclusion treatments in 2017, but even at the end of the growing season, achene weights were not statistically different among treatments, suggesting that biological significance is unlikely

We collected 142 achenes from 58 plants in 2014, 326 achenes from 116 plants in 2015, and 724 achenes from 73 plants in 2017. Achene weight did not change over the collection period (mean weight = 0.51 mg) and was unrelated to pollen treatment in 2014 (Table S4). Achene weight declined over the course of the season in 2015 (i.e., collection date was a significant covariate) but was unrelated to pollen treatment (Figure 7a; Table S4). In 2017, we saw a significant interaction between pollen treatment and achene collection date (Figure 7b, Table S4); however, at any given date (within the range of our collection dates), achene weight did not differ significantly among pollen treatments.

**FIGURE 8:**
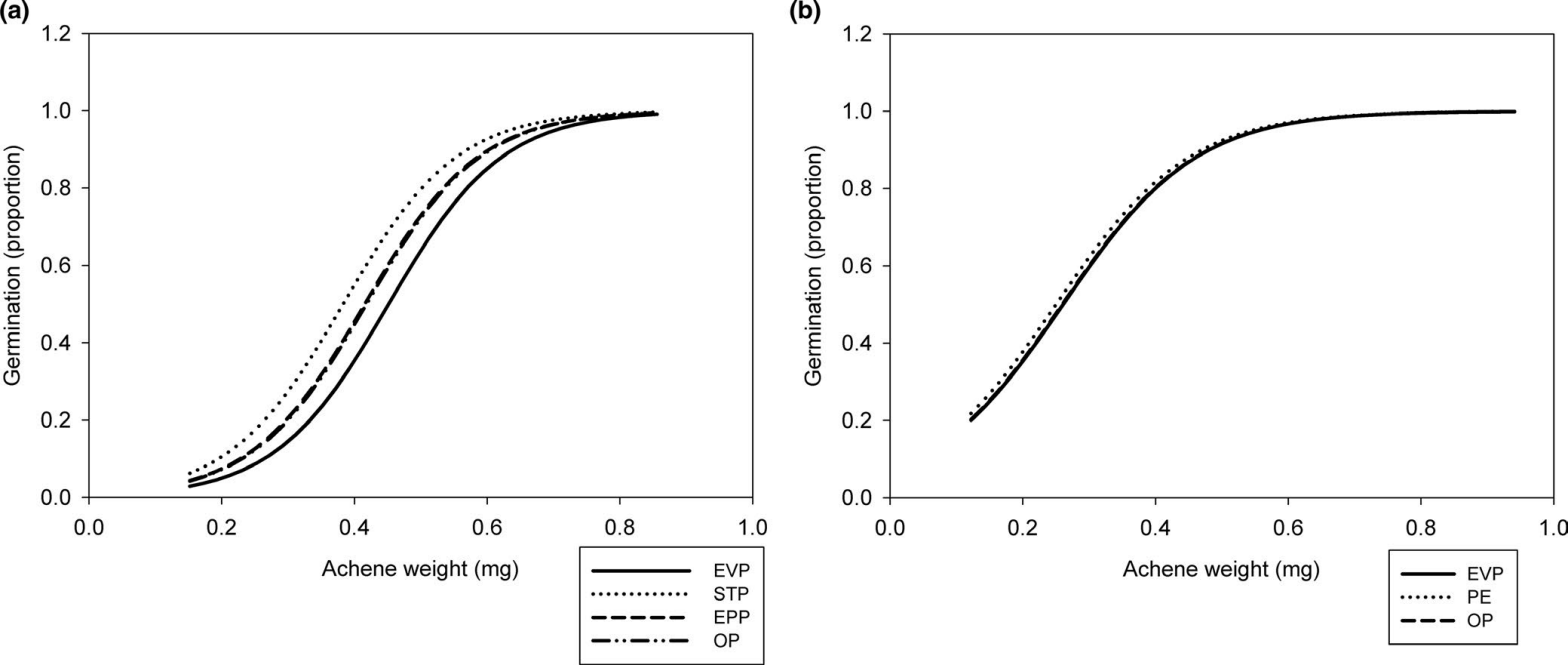
Proportion of *Eriogonum visheri* achenes that germinated in (a) 2015 and (b) 2017 as a function of achene weight and treatment. Abbreviations for treatments in the legends follow Table 1

We expected germination rate to be related to achene weight and collection date, so we used both as covariates to explain effects of pollination treatments on germination in 2015 and 2017. We did not assess germination rate in 2014, when we used collected achenes to develop a protocol to promote germination of ripe achenes. In 2015 and 2017, germination rate increased with achene weight, reaching an asymptote of near total germination in achenes weighing between 0.7 and 0.8 mg (Figure 8). In the 2015 and 2017 models, collection date was not significant and pollen treatment did not significantly affect germination rate in either year (Table S5). Data from the MOP treatment were too sparse (as described above), so were not evaluated in the model.

We found no evidence for pollen limitation due to autogamy in filled dehiscent achenes. Germination rate was slightly higher in PE than OP achenes: PL = 0.004 for germination of PE versus EVP achenes, and PL = −0.015 for germination of PE versus OP achenes. Based on the Baskin and Baskin (2018) standard, if PL falls between −0.01 and 0.01, germination is not pollen limited.

**FIGURE 9:**
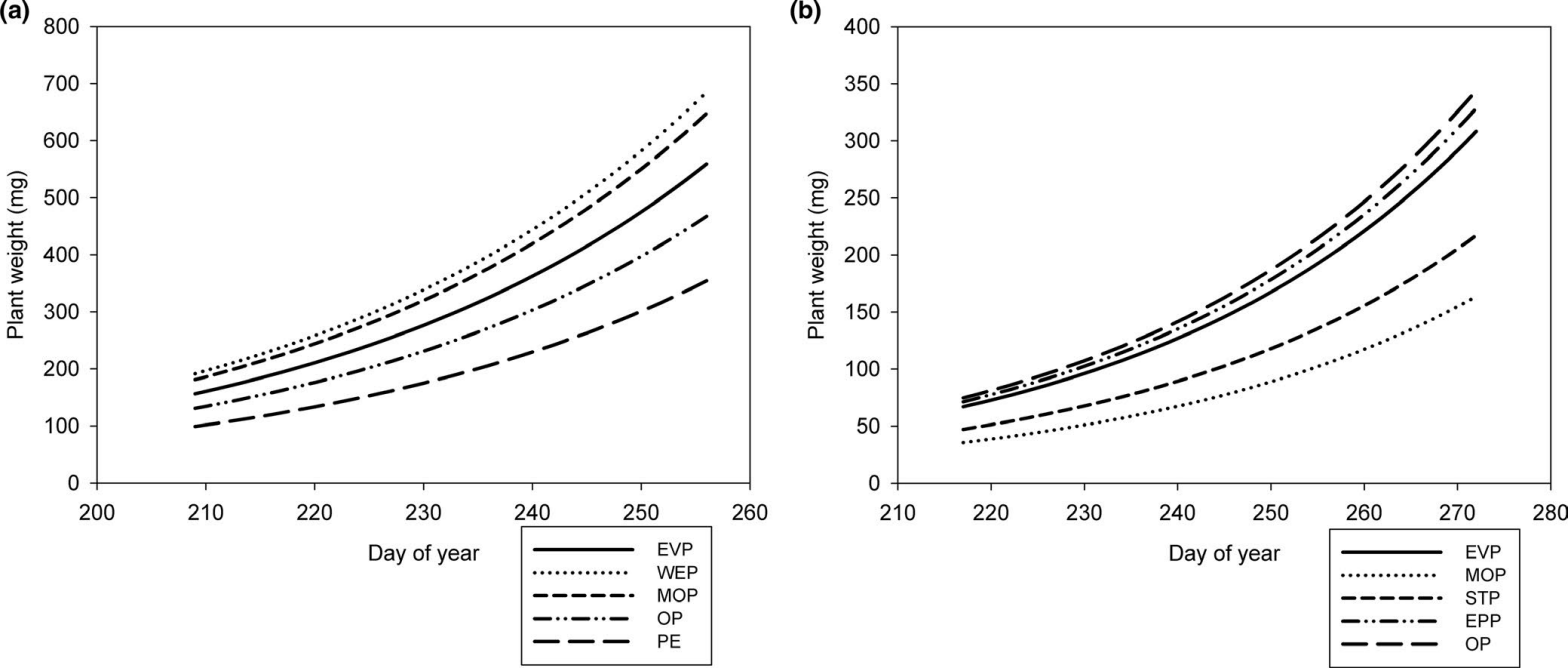
Air‐dried *Eriogonum visheri* plant weight in (a) 2014 and (b) 2015 as a function of the day‐of‐year that the plant was collected and treatment. Abbreviations for treatments in the legends follow Table 1

Plant weight did not vary among these treatments (Table S6), so effects we observed were not due to overall changes in plant vigor that depended on treatment. Plant biomass increased over time in 2014 and 2015 (Figure 9), but this effect was not significant in 2017 (Table S6). Plants were larger in 2014 than 2015, and average weight (113 mg) of plants in 2017 was smaller yet.

Data for all analyses presented in this paper are available online (Larson et al. 2021).

## 4 DISCUSSION

Many of the observations we made in this study were consistent with those in the network study (Larson et al., 2014). The striking lack of *M. officinalis* pollen on both insects and stigmas, despite super‐abundance in 2014, makes it clear that this invasive species has little chance of interfering with *E. visheri* pollination. Our data cannot conclusively exclude effects on total visits from potential pollinators, but the lack of differences in visitation to *E. visheri* among years, despite orders of magnitude more *M. officinalis* flowers in 2014, suggests neutrality. Flower morphology has been implicated in likelihood of pollinator (and pollen) sharing among coflowering species (Ashman et al., 2020) and this may play a part in the interaction between *E. visheri* and *M. officinalis*. Both species have small flowers, but *E. visheri's* are actinomorphic and *M. officinalis's* are zygomorphic. The small, open flowers of *E. visheri* may be especially accessible to the small halictids found in this study. Further, *M. officinalis* flowers are borne on tall, robust stems that can support insect visitors as large as bumble bees, while the fragile stems of *E. visheri* bow under the weight of most insects larger than halictids. A similar lack of overlap in flower visitors was found in a study of another rare plant, *Sidalcea hendersonii*, growing in the vicinity of invasive *Rubus armeniacus;* in that study, sweat bees were also the most common visitors to the rare species (Shelby & Peterson, 2015).

Despite earlier indications that *S. tragus* might interfere with *E. visheri* pollination as a result of shared pollinators (Larson et al., 2014), we found little evidence for this in the current study. Mean count of *S. tragus* pollen on collected stigmas was well under 1 grain per stigma. Likewise, pollen of the perennial congener, *E. pauciflorum*, averaged less than 1 grain per stigma. Importantly, neither pollen from the two invasive species nor from the congener significantly reduced achene weight when experimentally applied to *E. visheri* stigmas. This contrasts with the results of a meta‐analysis in which heterospecific pollen was more likely to reduce reproductive output of closely‐ than distantly related species (Arceo‐Gomez & Ashman, 2016). Our study did not evaluate total reproductive output, however, but only effects on dehiscent achenes. We found that achene weight was a good predictor of germination and the relationship did not vary with pollen treatment, further suggesting that *E. visheri* is not likely to suffer reduction in this fitness component due to heterospecific pollen receipt.

As in the case of Larson et al., (2014), *L. packeri* was the most common and consistent visitor to *E. visheri*, though other halictids sometimes carried more *E. visheri* pollen, especially in the long flowering season of 2015. The association between *E. visheri* and *L. packeri* is potentially an artifact of it being an abundant, generalist forager. However, *L. packeri* is the smallest bee caught in this study, (female body length 4.0–4.4 mm; (Gibbs, 2010)), so it may be a better size match with the small *E. visheri* flowers than other co‐occurring bees. Carrying more pollen likely indicates visits to more flowers and an increased likelihood of cross‐pollination. Based on germinability, *E. visheri* clearly is autogamous, but it lacks long‐distance seed dispersal mechanisms, a common adaptation in edaphically restricted plant species (Schenk, 2013), so may rely on pollinators for genetic diversity. Taking advantage of occasional advantageous conditions to extend the flowering season and attract a wider variety of pollinators may improve the chances of increasing genetic diversity via receiving pollen from a greater number of plants (Waananen et al., 2018). Studies of other habitat‐restricted plant populations, including a rare *Eriogonum* subspecies (Archibald et al., 2001) and several species restricted to gypsum outcrops (Matesanz et al., 2018), have not detected reduced genetic variation, which suggests the potential for adaptation to naturally fragmented habitats (Corlett & Tomlinson, 2020).

The decline in *E. visheri* pollen on the bodies of collected *L. packeri* and *P. haemorrhous* with increasing number of *E. visheri* flowers counted in 2015 was unexpected but may relate to the coflowering community. The number of *E. visheri* flowers was positively correlated with total flower counts, which may have reduced pollinator fidelity to *E. visheri* (Ashman et al., 2020). Indeed, nearly all of the recorded bee visitors are relatively small, generalist foragers (with the exception of one *Melissodes*, captured on *E. visheri* outside of a plot, which is an Asteraceae specialist and did not carry *E. visheri* pollen) that would not be expected to show any particular fidelity to *E. visheri*. Similarly diverse and unspecialized flower‐visitor assemblages were found for *Eriogonum pelinophilum*, a rare perennial buckwheat found in restricted habitats in western Colorado (Tepedino et al., 2011). Further, the numerous males collected on *E. visheri* would have primarily been visiting the flowers for nectar and would be expected to carry less pollen than females (Larson et al., 2018). Such interactions involving changes to the surrounding flowering plant community can be complex and dependent on pollinator community composition as well as changes in disturbance pattern and intensity (Portman et al., 2019).

Despite the presence of considerable amounts of invasive *S. tragus* pollen recovered from insects’ bodies, especially in 2015, little of this pollen was found on *E. visheri* stigmas. Similar observations were made by Bartomeus et al., (2008) in a study of invasive pollen transfer to native plants growing within *Carpobrotus* patches; they reasoned that lack of pollen dispersal to native stigmas could be tied to timing of stigma receptivity, floral constancy in individual pollinators, or location of the pollen on the insect's body. Of these, the most likely to be pertinent in our study is differences in pollen location on the insects’ bodies; we did not separate pollen from different body locations, so cannot test this hypothesis, but *S. tragus* flowers differ morphologically from those of *E. visheri*, and thus anthers may deposit pollen on sites unlikely to contact *E. visheri* stigmas. In contrast, all insects were generalists and were captured on *E. visheri* flowers, so constancy is an unlikely explanation. Similarly, the presence of pollen on nearly all collected stigmas (3.3% of 807 collections had no pollen) suggests the vast majority were receptive prior to collection. Stiers and Triest (2017), using fluorescent dyes as pollen analogues, found that pollen movement from an invasive species to a native was greatest in the immediate vicinity of the invasive. Given the patchy nature of *S. tragus* at our study sites, pollen may be lost passively or via grooming before the insect reaches many *E. visheri* flowers. Disentangling these hypotheses would require targeted experiments that we did not perform.

Mean achene weight declined over the collection period for all pollen treatments in 2015 but was constant in the shorter 2014 season and inconsistent but did not vary among pollen treatments in the still shorter 2017 season. The decline in 2015 seems unlikely to be related to pollination, as we saw no variation in the amount of pollen carried by insects over the flowering season. A more likely explanation is reduced nutrients and/or water as the season progressed, as precipitation in July—September was only 21% of that in April—June. Results of our germination studies suggest that these smaller achenes that ripened at the end of the season may be less likely to germinate, though this could be due to reduced viability or increased dormancy (Baskin, Chesson & Baskin 1993; Baskin & Baskin, 2014), which we did not test.

Although this study was not designed to test theories of germination as observed in *E. visheri*, we can add to the accumulating literature on conditions that favor *Eriogonum* germination (e.g., Young, 1989; Baskin, Chesson & Baskin 1993; Meyer & Paulsen, 2000). In particular, chilling alone was not effective at stimulating germination, at least within the refrigerator and greenhouse conditions our achenes experienced. However, scarification of the tip of the achene after chilling during the year after collection did result in high germination rates, given the temperature and light conditions of our greenhouse. As Baskin, Chesson and Baskin (1993) pointed out for the winter annual *Eriogonum abertianum*, the predictability of the environment will likely influence the degree and kind of dormancy annuals will display. In the case of *E. visheri*, environmental events such as freeze‐thaw cycles may result in abrasion, which, combined with imbibing, may signal suitable conditions for germination.

More than a decade ago Mitchell et al., (2009) proposed a framework for mechanisms of competition for pollination that traced visit number and quality through (for females) pollen quantity, pollen quality, and heterospecific pollen receipt, to the number of seeds produced as an indicator of fitness. Applying this framework to what we have now learned about the rare endemic, *E. visheri*, several aspects of its life history point to adaptation to rarity. The amount and quality (i.e., originating from a different conspecific plant) of pollen brought to a stigma by pollinators, though undoubtedly important for genetic diversity, does not control seed production in this self‐compatible, autogamous annual. Likewise, within the limits of our experimental protocol, heterospecific pollen on the stigma does not reduce achene weight or germination compared to any other treatment, including open or augmented conspecific pollination. While discussions of species rarity often focus on causes of rarity (e.g., Kunin & Gaston, 1993; Walck, Baskin & Baskin 2001; Combs et al., 2013), *E. visheri* may rather provide an example of a species well adapted to rarity.

## 5 CONCLUSION

Management implications based on the network analysis were only half right. *Melilotus officinalis* is not a threat to pollination of *E. visheri*, but neither is *S. tragus*. Although we found some *E. pauciflorum* pollen on stigmas of *E. visheri*, we have no evidence that its pollen is detrimental to *E. visheri* reproduction. Indeed, the fact that we found small amounts of *E. pauciflorum* pollen on 22% of *E. visheri* stigmas suggests the abundant and perennial *E. pauciflorum* provides alternative resources to *E. visheri's* pollinators. de Santiago‐Hernandez et al., (2019) urged caution in interpretation of visitation network results, having found that when they excluded visits that did not lead to seed set, network metrics changed dramatically. Our results suggest that, from the plant's perspective, details such as autogamy and effects of heterospecific pollen are key factors not accounted for in network analysis. Nonetheless, the network analysis upon which this study was based did provide knowledge of the pertinent community of plants and insects, if not the specifics and direction of each interaction.

Larson et al., (2014) contrasted the broad, community‐level understanding gained with a network approach with more limited, but more specific, understanding gained in a study directed at the focal plant's visitors. In this case, we gained more confidence in the effects of the invasive species by directly assessing effects of their pollen on achene size and germination. Although it was still necessary to collect insect visitors for identification, fewer individuals were involved. In addition, the current study added new insight into germination requirements for a rare, edaphically specialized species of *Eriogonum*.

## CONFLICTS OF INTEREST

None declared.

## AUTHORS’ CONTRIBUTIONS

DLL, JLL, and AJS: Conception and design study and carry out the field work. ZMP: Identification of insects collection JLL: Identification of pollen. JLL and ZMP: Data curation. DAB, DLL, AJS, and JLL: Participation in data analysis. All authors: Contribution in writing the original draft manuscript and revisions; and final approval for publication.

## Supporting information

Supplementary Material

## Data Availability

Data supporting the results: ScienceBase.gov, https://doi.org/10.5066/P9MNIVB3.
