# Supplementary material for: Coflowering invasive plants and a congener have neutral effects on fitness components of a rare endemic plant
Source: Ecol Evol. 2021 Mar 20;11(9):4750–62. doi: 10.1002/ece3.7375 (PMC8093677; doi:10.1002/ece3.7375)
Supplement: Supplementary file 1 — Supplementary Material [file ECE3-11-4750-s001.docx]

Table S1. Counts of aborted and filled achenes of *Eriogonum visheri* in 2014 and 2015. Germination tests were not conducted in 2014 and aborted achenes were not counted in 2017.

| Year | Treatment^a^ | Aborted achenes | Filled achenes | Total aborted + filled achenes | Filled achenes (% of Total) | Germinated achenes (% of Total) |
| --- | --- | --- | --- | --- | --- | --- |
| 2014 | EVP | 24 | 15 | 39 | 38 |  |
|  | PE | 21 | 23 | 44 | 52 |  |
|  | MOP | 8 | 5 | 13 | 38 |  |
|  | OP | 49 | 65 | 114 | 57 |  |
|  | WEP | 26 | 32 | 58 | 55 |  |
|  | Grand Total | 120 | 135 | 255 | 53 |  |
| 2015 | EVP | 67 | 89 | 156 | 57 | 75 |
|  | EPP | 24 | 42 | 66 | 64 | 79 |
|  | MOP | 15 | 16 | 31 | 52 | 78 |
|  | OP | 66 | 117 | 183 | 64 | 80 |
|  | SIP | 68 | 57 | 125 | 46 | 73 |
|  | Grand Total | 240 | 321 | 561 | 57 | 77 |

^a^ EVP = *E. visheri* pollen from 3 other plants added; PE = pollinators excluded; MOP = *Melilotus officinalis* + *E. visheri* pollen added; OP = open pollinated; WEP = *E. visheri* pollen from 3 flowers on the same plant added; EPP = *E. pauciflorum* and *E. visheri* pollen added; SIP = *Salsola tragus* + *E. visheri* pollen added.

Table S2. Number of stigmas collected from *Eriogonum visheri* plants during each week of the study in 2014, 2015 and 2017.

| Year | Week | Plants sampled (N) | Stigmas (N) |
| --- | --- | --- | --- |
| 2014 | 26 | 2 | 3 |
|  | 27 | 14 | 35 |
|  | 28 | 340 | 946 |
|  | 29 | 45 | 120 |
|  | 30 | 30 | 85 |
|  | 31 | 12 | 35 |
|  | 32 | 6 | 18 |
|  | 33 | 6 | 17 |
| 2015 | 26 | 1 | 3 |
|  | 27 | 8 | 22 |
|  | 28 | 10 | 24 |
|  | 29 | 46 | 122 |
|  | 30 | 16 | 39 |
|  | 31 | 39 | 93 |
|  | 32 | 42 | 114 |
|  | 33 | 31 | 87 |
|  | 34 | 38 | 99 |
|  | 35 | 35 | 95 |
|  | 36 | 22 | 63 |
| 2017 | 28 | 1 | 3 |
|  | 33 | 44 | 128 |
|  | 34 | 11 | 30 |
|  | 36 | 8 | 23 |
| Total |  | 807 | 2204 |

Table S3. Insects captured on *Eriogonum visheri* flowers and the number of pollen grains of the focal species and total pollen grains removed from the insects’ bodies. Pollen values shown are mean + SE per captured insect.

| Year | Insect type | Insect species | N | *E. visheri* | *E. pauciflorum* | *Melilotus officinalis* | *Salsola tragus* | Total pollen |
| --- | --- | --- | --- | --- | --- | --- | --- | --- |
| 2014 | Ants | Ant, unidentified sp. | 6 | 9+6 | 18+7 | 0 | 0 | 27+ 11 |
|  | Bees | *Lasioglossum packeri* | 11 | 752+263 | 108+33 | 0 | 567+ 406 | 1426+634 |
|  | Bees | *Lasioglossum semicaeruleum* | 1 | 0 | 0 | 600 | 0 | 600 |
|  | Flies | Fly, unidentified sp. | 5 | 12+8 | 2+1 | 2+ 2 | 0 | 16+ 10 |
|  | Flies | *Paragus haemorrhous* | 9 | 2+2 | 0 | 0 | 0 | 2+ 2 |
|  | Flies | Syrphid, unidentified sp. | 17 | 1+1 | 0 | 0 | 0 | 1+ 1 |
|  | Wasps | Wasp, unidentified sp. | 3 | 418+410 | 42+24 | 0 | 0 | 460+ 444 |
| 2015 | Ants | Ant, unidentified sp. | 3 | 0 | 0 | 0 | 0 | 0 |
|  | Bees | *Agapostemon angelicus* | 1 | 41 | 0 | 0 | 0 | 41 |
|  | Bees | *Halictus confusus* | 14 | 140+67 | 109+29 | 0 | 61+22 | 356+169 |
|  | Bees | *Halictus tripartitus* | 3 | 38+23 | 0 | 0 | 154+105 | 238+ 142 |
|  | Bees | *Lasioglossum occidentale* | 13 | 22+8 | 1+0.4 | 0 | 4+2 | 34+ 13 |
|  | Bees | *Lasioglossum packeri* | 46 | 201+73 | 6+1 | 0 | 232+127 | 452+146 |
|  | Bees | *Lasioglossum semicaeruleum* | 2 | 881+822 | 0 | 0 | 1259+1243 | 2140+2066 |
|  | Bees | *Lasioglossum trigeminum* | 8 | 1564+959 | 0 | 0 | 253+214 | 1819+ 968 |
|  | Beetles | Beetle, unidentified sp. | 1 | 0 | 0 | 0 | 0 | 0 |
|  | Flies | Fly, unidentified sp. | 8 | 3+2 | 0 | 0 | 9+9 | 12+ 9 |
|  | Flies | *Paragus haemorrhous* | 27 | 61+46 | 2+0.4 | 0 | 6+5 | 69+51 |
|  | Wasps | Wasp, unidentified sp. | 5 | 14+7 | 3+2 | 0 | 0 | 20+11 |
| 2017 | Bees | *Lasioglossum packeri* | 12 | 78+35 | 12+3 | 0 | 206+141 | 298+137 |
| Grand Total | |  | 195 | 198+51 | 18+1 | 3+3 | 131+42 | 356+74 |

Table S4. Type III tests of fixed effects for effect of pollen treatment on achene weight of *Eriogonum visheri*, with achene collection date as a covariate, by year. Full model is the initial model with test for covariate interaction; final model shows effects after removal of non-significant covariate interaction. P-values in bold in the final models are significant at P<0.05.

| Year | Model | Effect | Num DF | Den DF | F Value | Pr > F |
| --- | --- | --- | --- | --- | --- | --- |
| 2014 | Full | Pollen treatment | 4 | 11 | 1.59 | 0.2445 |
|  |  | Achene collection date | 1 | 76 | 1.89 | 0.1733 |
|  |  | Achene collection date*treatment | 4 | 76 | 1.48 | 0.2178 |
|  |  |  |  |  |  |  |
|  | Final | Pollen treatment | 4 | 12 | 1.86 | 0.1820 |
|  |  | Achene collection date | 1 | 79 | 0.21 | 0.6461 |
|  |  |  |  |  |  |  |
| 2015 | Full | Pollen treatment | 4 | 6 | 2.13 | 0.1949 |
|  |  | Achene collection date | 1 | 194 | 35.30 | <.0001 |
|  |  | Achene collection date*treatment | 4 | 194 | 2.20 | 0.0703 |
|  |  |  |  |  |  |  |
|  | Final | Pollen treatment | 4 | 6 | 0.70 | 0.6196 |
|  |  | Achene collection date | 1 | 198 | 29.50 | **<.0001** |
|  |  |  |  |  |  |  |
| 2017 | Full/Final | Pollen treatment | 2 | 2 | 3.45 | 0.2246 |
|  |  | Achene collection date | 1 | 498 | 0.64 | 0.4256 |
|  |  | Achene collection date*treatment | 2 | 498 | 3.35 | **0.0358** |

Table S5. Type III tests of fixed effects for effect of pollen treatment on proportion of *Eriogonum visheri* achenes that germinated, with achene weight and achene collection date as covariates, by year. Germination was not assessed in 2014. Full model is the initial model with test for covariate interactions; interim models with non-significant covariates removed sequentially are followed by the final model, which shows effects (P values in bold are significant at P<0.05) after removal of all non-significant covariate effects.

| Year | Model | Effect | Num DF | Den DF | F Value | Pr > F |
| --- | --- | --- | --- | --- | --- | --- |
| 2015 | Full | Pollen treatment | 3 | 6 | 0.4 | 0.7565 |
|  |  | Achene collection date | 1 | 182 | 1.8 | 0.1817 |
|  |  | Achene collection date * treatment | 3 | 182 | 0.43 | 0.7339 |
|  |  | Achene weight | 1 | 182 | 30.64 | <.0001 |
|  |  | Achene weight*treatment | 3 | 182 | 1.01 | 0.388 |
|  | Interim | Pollen treatment | 3 | 6 | 0.98 | 0.4605 |
|  |  | Achene collection date | 1 | 185 | 1.28 | 0.259 |
|  |  | Achene weight | 1 | 185 | 36.27 | <.0001 |
|  |  | Achene weight*treatment | 3 | 185 | 0.99 | 0.3973 |
|  | Interim | Pollen treatment | 3 | 6 | 0.43 | 0.7365 |
|  |  | Achene collection date | 1 | 188 | 1.05 | 0.3068 |
|  |  | Achene weight | 1 | 188 | 55.06 | <.0001 |
|  | Final | Pollen treatment | 3 | 6 | 0.54 | 0.6741 |
|  |  | Achene weight | 1 | 190 | 58.77 | **<.0001** |
|  |  |  |  |  |  |  |
| 2017 | Full | Pollen treatment | 2 | 2 | 1.26 | 0.4425 |
|  |  | Achene collection date | 1 | 467 | 0.82 | 0.3665 |
|  |  | Achene collection date * treatment | 2 | 467 | 1.01 | 0.3664 |
|  |  | Achene weight | 1 | 467 | 18.44 | <.0001 |
|  |  | Achene weight*treatment | 2 | 467 | 0.74 | 0.4798 |
|  | Interim | Pollen treatment | 2 | 2 | 1.42 | 0.4134 |
|  |  | Achene collection date | 1 | 469 | 0.59 | 0.4414 |
|  |  | Achene collection date * treatment | 2 | 469 | 1.42 | 0.2416 |
|  |  | Achene weight | 1 | 469 | 44.57 | <.0001 |
|  | Interim | Pollen treatment | 2 | 2 | 0.01 | 0.9881 |
|  |  | Achene collection date | 1 | 471 | 0.03 | 0.8719 |
|  |  | Achene weight | 1 | 471 | 44.61 | <.0001 |
|  | Final | Pollen treatment | 2 | 2 | 0.01 | 0.9901 |
|  |  | Achene weight | 1 | 472 | 45.32 | **<.0001** |

Table S6. Type III tests of fixed effects for effect of pollen treatment on air-dried plant weight of *Eriogonum visheri*, with plant collection date as a covariate, by year. Full model is the initial model with test for covariate interaction; final model shows effects after removal of non-significant covariate interaction. P-values in bold in the final model are significant at P<0.05.

| Year | Model | Effect | Num DF | Den DF | F Value | Pr > F |
| --- | --- | --- | --- | --- | --- | --- |
| 2014 | Full | Pollen treatment | 4 | 12 | 0.4 | 0.8023 |
|  |  | Plant collection date | 1 | 58 | 9.07 | 0.0038 |
|  |  | Plant collection date*treatment | 4 | 58 | 0.34 | 0.8488 |
|  |  |  |  |  |  |  |
|  | Final | Pollen treatment | 4 | 12 | 0.99 | 0.4485 |
|  |  | Plant collection date | 1 | 62 | 10.26 | **0.0021** |
|  |  |  |  |  |  |  |
| 2015 | Full | Pollen treatment | 4 | 6 | 1.06 | 0.4525 |
|  |  | Plant collection date | 1 | 118 | 20.01 | <.0001 |
|  |  | Plant collection date*treatment | 4 | 118 | 0.96 | 0.4306 |
|  |  |  |  |  |  |  |
|  | Final | Pollen treatment | 4 | 6 | 1.54 | 0.3033 |
|  |  | Plant collection date | 1 | 122 | 21.45 | **<.0001** |
|  |  |  |  |  |  |  |
| 2017 | Full | Pollen treatment | 2 | 1 | 0.11 | 0.9026 |
|  |  | Plant collection date | 1 | 12 | 0.04 | 0.8445 |
|  |  | Plant collection date*treatment | 2 | 12 | 0.12 | 0.8898 |
|  |  |  |  |  |  |  |
|  | Final | Pollen treatment | 2 | 2 | 0.45 | 0.6896 |
|  |  | Plant collection date | 1 | 13 | 0.22 | 0.647 |

Figure S1. Mean + standard error of total flower counts/year for all flowering forbs, excluding *M. officinalis* (a) and including *M. officinalis* (b). Note that Y-axes differ between panels.
